# Supplementary material for: Southern Hemisphere westerlies as a driver of the early deglacial atmospheric CO2 rise
Source: Nat Commun. 2018 Jun 27;9:2503. doi: 10.1038/s41467-018-04876-4 (PMC6021399; doi:10.1038/s41467-018-04876-4)
Supplement: Supplementary file 1 — Supplementary Information [file 41467_2018_4876_MOESM1_ESM.pdf]

Supplementary Information: Southern Hemisphere westerlies as a driver of the early deglacial atmospheric CO_2 rise

L. Menviel [1,2*], J.P. Spence [1], J. Yu [3], M. Chamberlain [4], R. Matear [4], K.J. Meissner [1],M.H. England [1]

[1]*Climate Change Research Centre and ARC Centre of Excellence for Climate System Science, University of New South Wales, Sydney, Australia*

[2]*Department of Earth and Planetary Sciences, Macquarie University, Sydney, Australia*

[3]*Research School of Earth Sciences, The Australian National University, Canberra, Australia*

[4]*CSIRO Oceans and Atmosphere, Hobart, Australia*

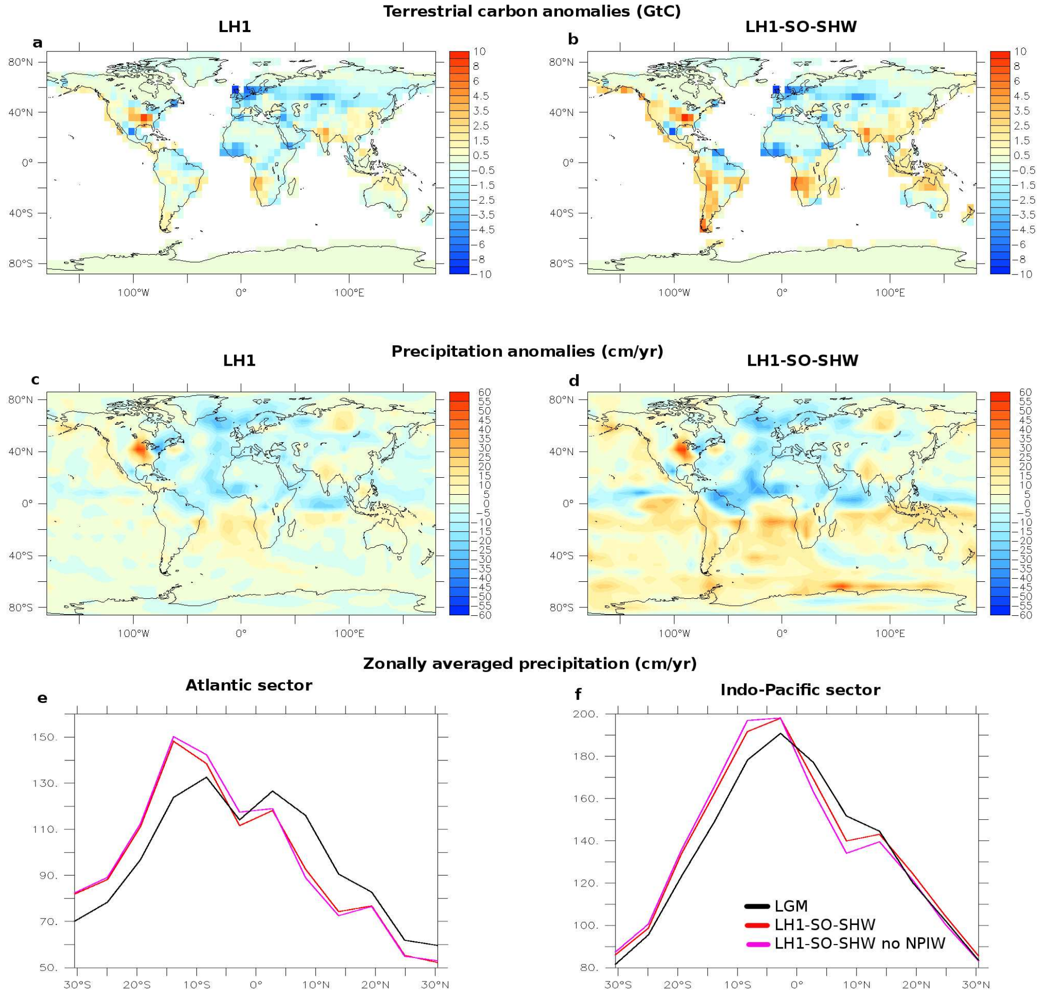

Supplementary Fig. 1: **a-b, Terrestrial carbon (GtC) and c-d, precipitation anomalies (cm yr^{-1})** as simulated in **a, c,** LH1 and **b, d,** LH1-SO-SHW at 16 ka compared to 19 ka. **e, f,** Total precipitation (cm yr^{-1}) zonally averaged over **e,** the Atlantic sector (80°W-20°E) and **f,** the Indo-Pacific sector (50°E-260°E) for the LGM control run (black), simulations LH1-SO-SHW (red) and LH1-SO-SHW with cessation of NPIW (magenta) averaged over years 16.0 - 15.8 ka.

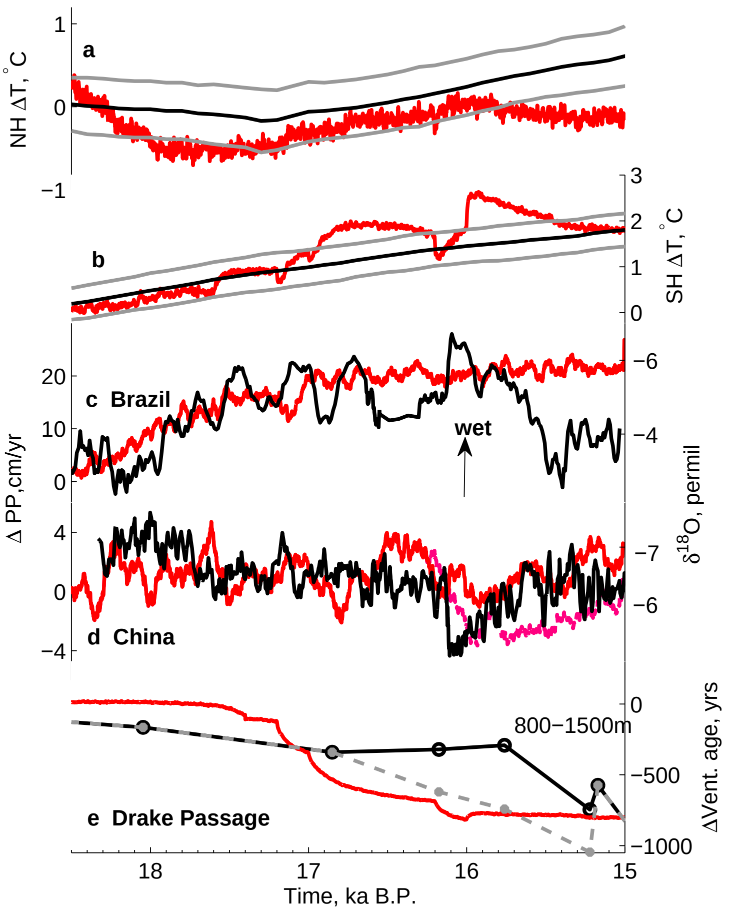

Supplementary Fig. 2: **Simulated changes across HS1 (LH1-SO-SHW, red) compared to paleoproxy records (black).** Air temperature anomalies (°) globally averaged over **a,** the Northern Hemisphere and **b,** the Southern Hemisphere, compared to an estimate based on proxy records[1]; **c,** Annual mean precipitation anomalies (45°W-37°W, 9°S-16°S) compared to a δ^{18}O record from Paixão cave, Brazil[2]; **d,** Boreal summer precipitation anomalies (98°W-110°W, 14°N-34°N) compared to a stack of δ^{18}O records from Qingtian and Hulu caves, China[3]; The pink dashed line includes cessation of NPIW formation at 16.2 ka. **e,** Changes in ventilation ages (years) in the Southern Ocean (75°S-50°S, 700-1000 m depth), compared to an estimate based on coral records from the Drake Passage[4,5]. The reference for ventilation ages anomalies are LGM (18–20 ka) values. The Drake Passage deep sea corals were taken from depth ranging from 800 to 1500 m, with LGM (18–20 ka) corals only originating from ∼800 m, while corals dated at 16.176 ka and 15.221 ka are from ∼1200 m depth and the coral dated at 15.761 ka is from 1516 m depth. Given that the ventilation age of waters increases with depth, this difference in water depth has to be taken into account. The grey line thus adds an age offset to the coral data based on their water depth and on the simulated mean ventilation age difference with depth (i.e. 300 years was added to corals from ∼1200 m and 450 years for the coral from 1516 m depth).

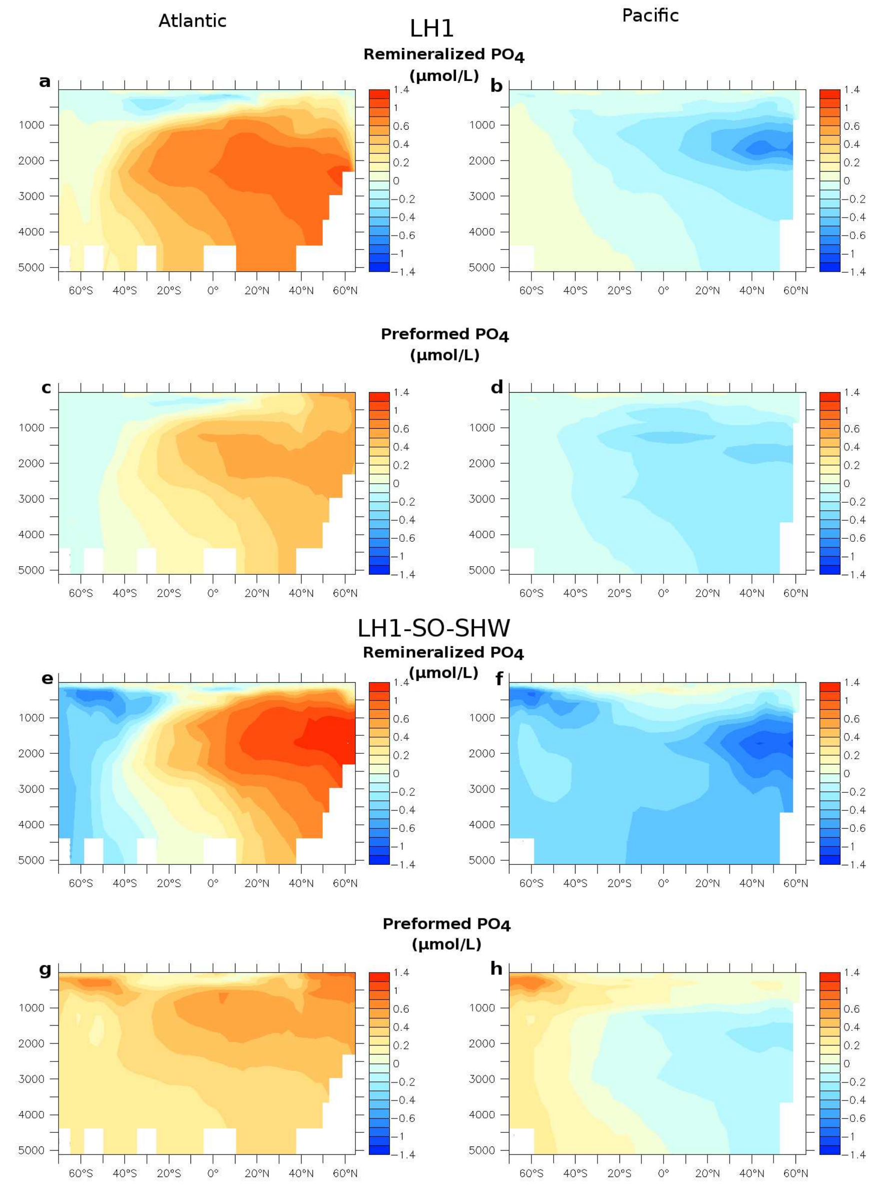

Supplementary Fig. 3: Changes in **a-b, e-f,** remineralized and **c-d, g-h,** preformed PO_4 (μmol L^{-1}) zonally averaged over (left) the Atlantic and (right) the Pacific, for experiments **a-d,** LH1 and **e-h,** LH1-SO-SHW at 15 ka compared to 19 ka. Phosphate (PO_4) content is separated into PO_{4Rem} and PO_{4Pref}: $PO_4 = PO_{4Rem} + PO_{4Pref}$ with PO_{4Rem} = AOU*$R_{P/O}$ and AOU = O_{2sat} - O_2. $R_{P/O}$ represents the Redfield ratio of P/O; AOU is the Apparent Oxygen Utilization and O_{2sat} the dissolved oxygen content based on temperature and salinity.

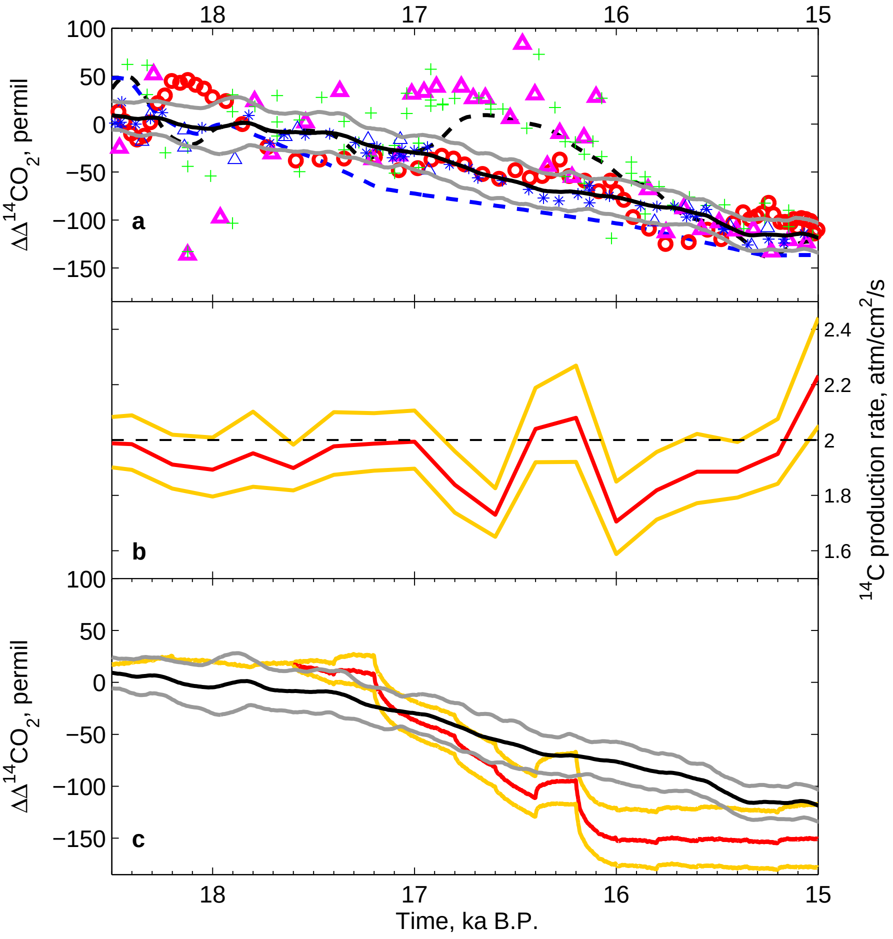

Supplementary Fig. 4: Δ^{14}C **reconstructions and influence of production rate on atmospheric** Δ^{14}**C.** Timeseries of **a,** Estimated atmospheric Δ^{14}C anomalies (‰): Intcal products for year 2013 (black)[6] with 2σ uncertainty (grey), 2009 (dashed black)[7] and 2004 (dashed blue)[8]; from Lake Suigetsu, Japan, (red circles)[9]; based on marine sediment core from the Cariaco Basin (magenta trianles)[10] and (green crosses)[11]; based on Hulu cave speleothem (blue stars and triangles)[12]. **b,** atmospheric ^{14}C production rate as estimated in Hain et al. (2014) [13] **c,** simulated atmospheric Δ^{14}C anomalies in experiment LH1-SO-SHW forced with the mean (red) as well as minimum and maximum (orange) ^{14}C production rates shown in **a,**. The IntCal 2013 atmospheric Δ^{14}C value[6] is shown in black with 2σ uncertainty (grey).

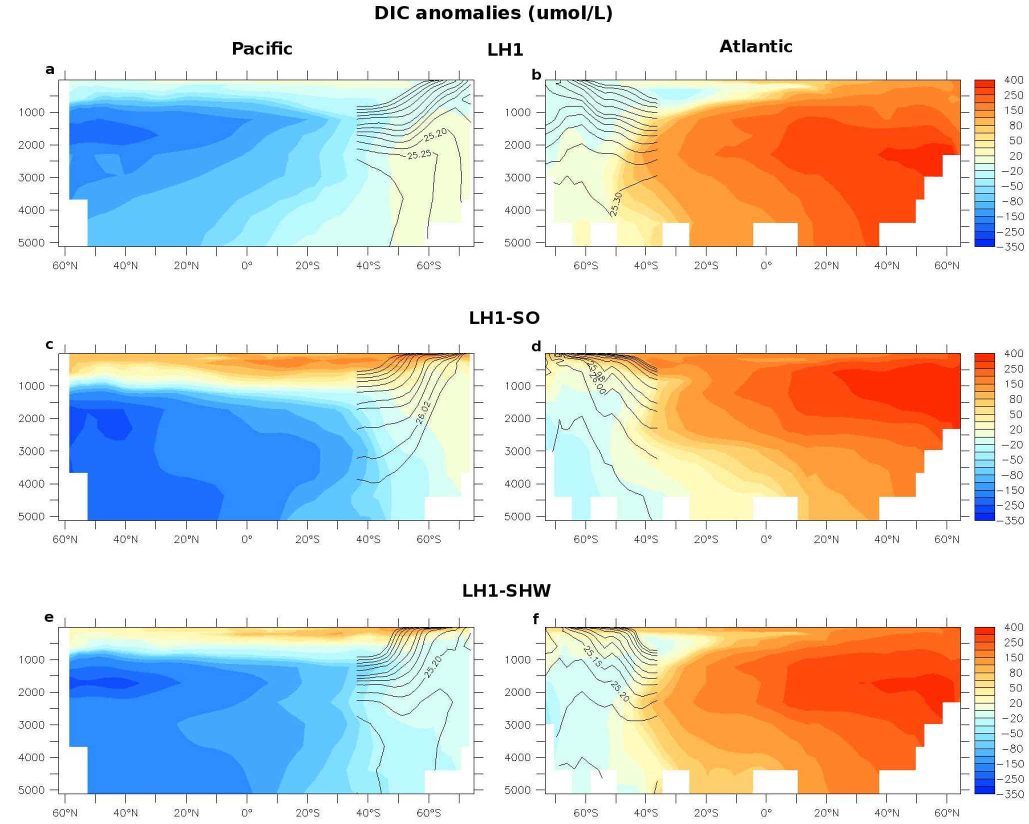

Supplementary Fig. 5: **HS1 DIC anomalies** (μmol L^{-1}) as simulated at 16 ka in experiments **a,b,** with weak AABW and AAIW (LH1), **c,d,** strong AABW but weak AAIW resulting from weak SH westerlies (LH1-SO), and **e,f,** strong SH westerlies but with a background LGM Southern Ocean freshwater flux (LH1-SHW). DIC anomalies are zonally averaged over (left) the Pacific and (right) the Atlantic basin and compared to DIC values at 19 ka. The Southern Ocean isopycnals are overlaid.

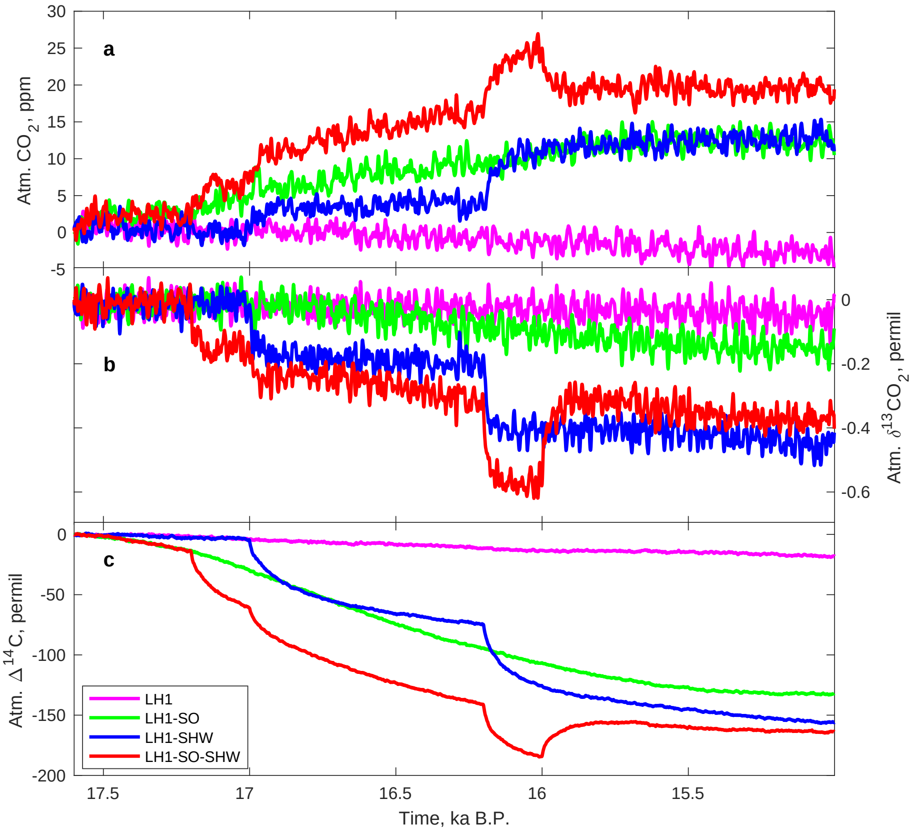

Supplementary Fig. 6: **Simulated atmospheric CO_2 concentration, $\delta^{13}CO_2$ and atmospheric $\Delta^{14}C$.** Time-series of **a,** atmospheric CO_2, **b,** $\delta^{13}CO_2$ and **c,** atmospheric $\Delta^{14}C$ anomalies as simulated in LH1 (magenta), LH1-SO (green), LH1-SHW (blue) and LH1-SO-SHW (red).

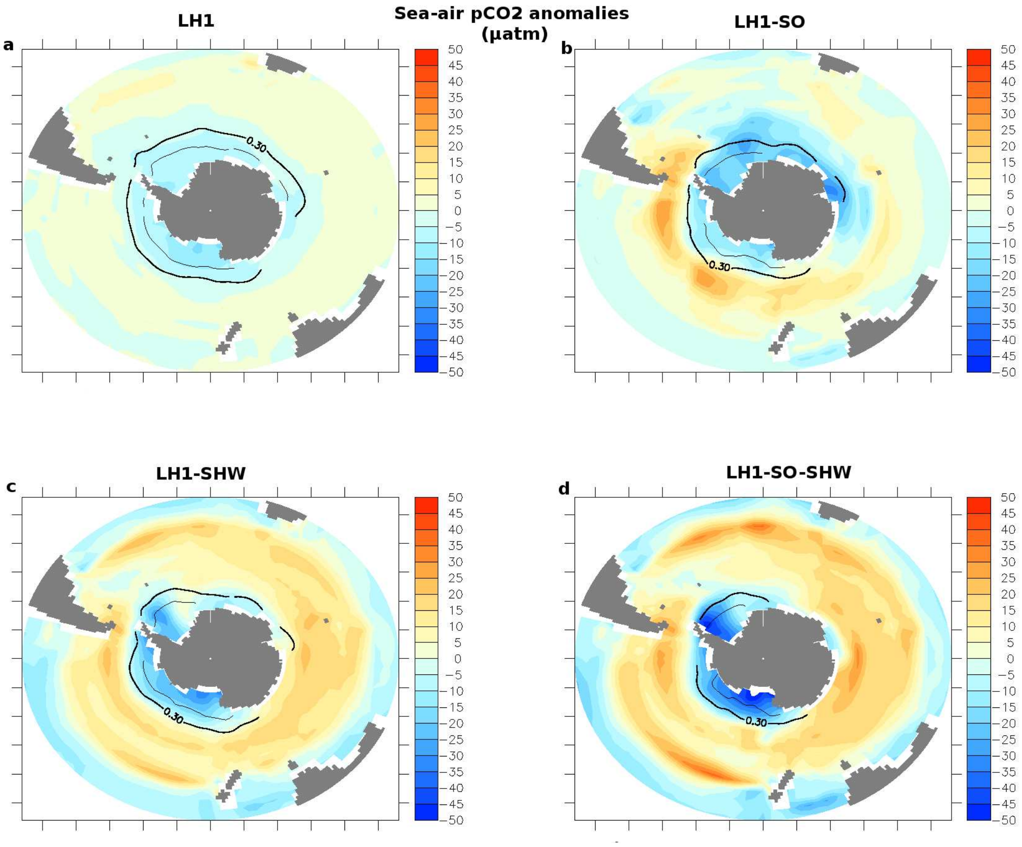

Supplementary Fig. 7: **Sea-air pCO$_2$ anomalies** (μatm) as simulated in **a,** LH1, **b,** LH1-SO, **c,** LH1-SHW and **d,** LH1-SO-SHW at 16 ka compared to 19 ka. Positive values indicate a potential CO_2 flux out of the ocean. The 30% (thick line) and 85% (thin line) austral summer sea-ice contour are overlaid.

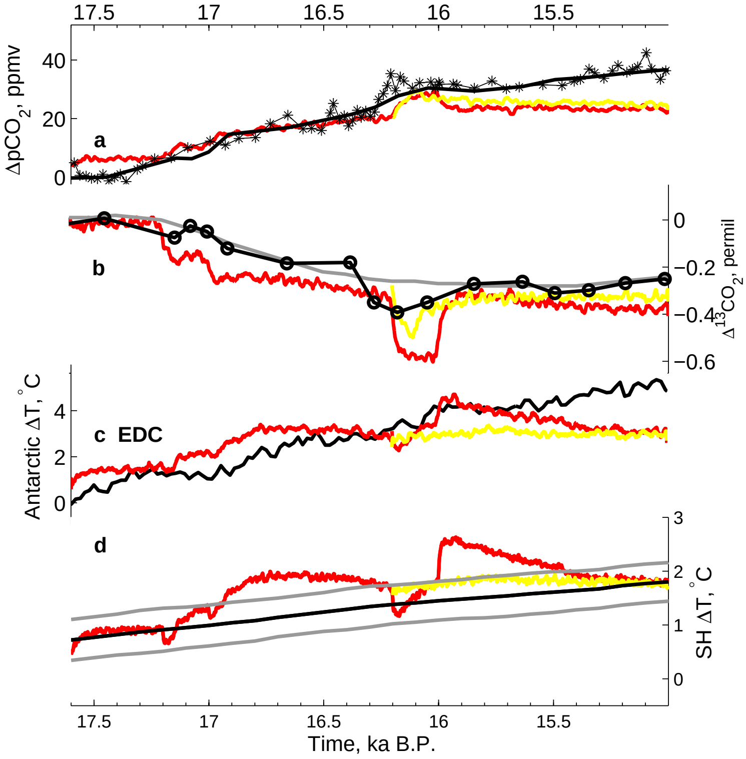

Supplementary Fig. 8: **Impact of a terrestrial carbon release at 16.2 ka.** Timeseries of **a,** atmospheric CO_2 anomalies (ppm) and **b,** $\delta^{13}CO_2$ anomalies (‰); Air temperature anomalies (°C) averaged over **c,** Antarctica and **d,** the Southern Hemisphere for simulation LH1-SO-SHW (red) and a simulation with an imposed 50 GtC terrestrial carbon release over 100 years (0.5 GtC yr^{-1}) at 16.2 ka and with a $\delta^{13}C$ signature of -24‰ (yellow). The simulations are compared to Antarctic ice core proxy data (black)[14–16] and an hemispheric temperature estimate[1].

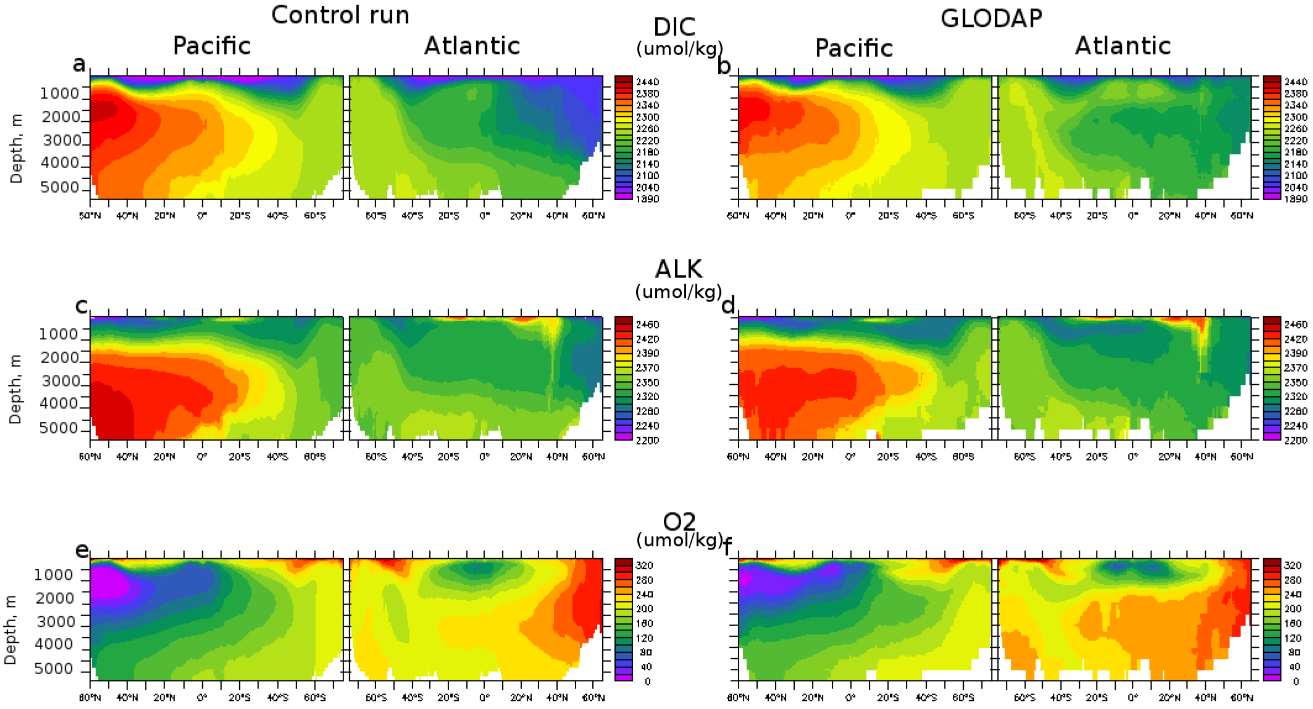

Supplementary Fig. 9: **Biogeochemical tracers distribution** (left) as simulated in the control state of the global eddy permitting ocean sea-ice carbon cycle model, and compared to (right) the observed GLODAPv2 dataset[17] for **a,b,** DIC (μmol kg^{-1}), **c,d,** alkalinity (μmol kg^{-1}), **e,f,** and dissolved oxygen (μmol kg^{-1}), zonally averaged over the Pacific and Atlantic basins.

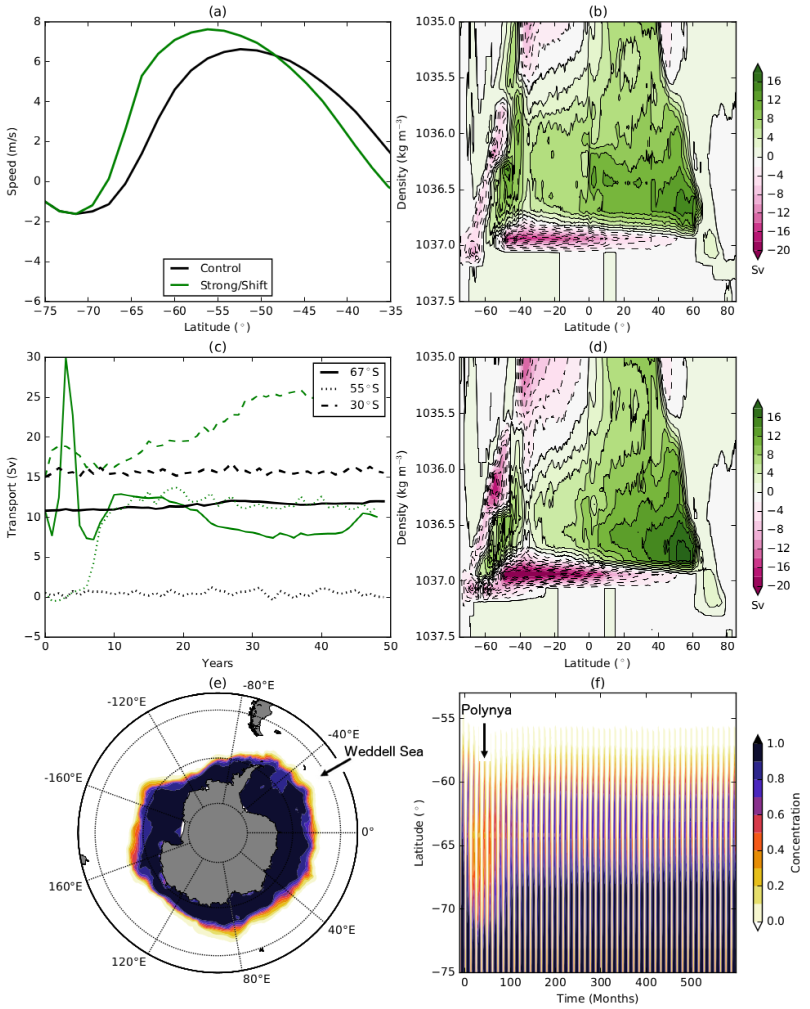

Supplementary Fig. 10: **Eddy permitting global ocean model response to poleward intensifying SH westerlies.** **a,** Annual mean, zonal average, zonal wind stress (N m^{-2}) in the (black) control state and (green) polar intensified wind perturbation experiment. **b,** Control state meridional overturning circulation transport (Sv) in density space (2000 m reference depth). **c,** Absolute annual mean transport (Sv) of the lower cell of the meridional circulation across various SH latitudes in (black) the control and (green) polar intensified case. **d,** Meridional overturning circulation transport (Sv) density space in the poleward intensified case and averaged over years 45-50. It shows an intensification of the bottom overturning cell (AABW) at a density of about 1037 kg m^{-3} and of AAIW at 1036-1036.5 kg m^{-3} (calculated relative to 2000 m reference depth) compared to the control run. **e,** July, August, September Southern Ocean sea ice concentration in the control run. **f,** Hovmöller diagram of monthly average sea ice concentration, zonally averaged between 60°W-20°W in the Weddell Sea region in the polar intensified wind experiment.

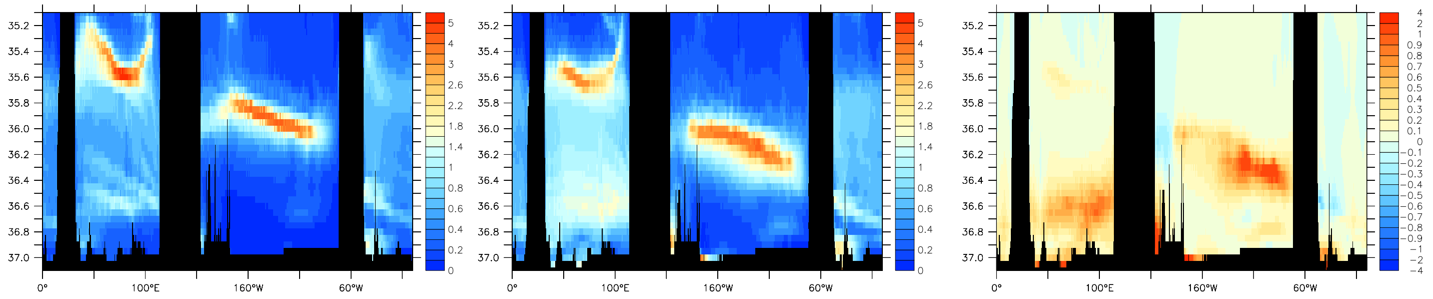

Supplementary Fig. 11: **Oceanic CFC-11 content in the global eddy permitting ocean sea-ice carbon cycle model** (x$10^{-6}\mu$mol L^{-1}) at 30°S as a function of seawater density (kg m^{-3}-1000, 2000m reference depth) for the (left) control run, (middle) the polar intensified westerly simulation and (right) the polar intensified westerly simulation at year 50 compared to the control run.

	Δ **Global Carbon** (GtC)			Δ **Deep Ocean** (GtC)			
Experiment	Ocean	Terr.	Atm.	Δ Atl.	Δ Pac.	Δ Ind.	Δ SO
LH1	11	-10	-1	303	-253	7	23
LH1-SO	-54	23	31	151	-499	-40	-40
LH1-SHW	-76	43	33	240	-358	-22	-26
LH1-SO-SHW	-99	52	47	179	-478	-40	-46

Supplementary Table 1: **Changes in oceanic, terrestrial and atmospheric carbon reservoirs** (GtC) between 15 and 19 ka B.P. for the different experiments performed. Changes in deep ($\geq$2000 m) ocean carbon reservoirs are further separated into Atlantic, Pacific and Indian and Southern Ocean (SO) components.

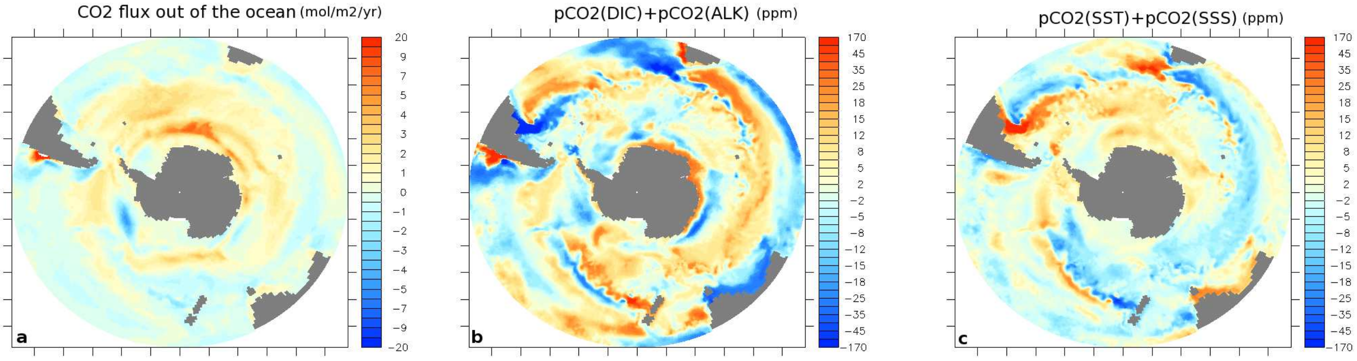

Supplementary Fig. 12: **CO_2 flux in the global eddy permitting ocean sea-ice carbon cycle model. a,** Anomalies in CO_2 flux out of the ocean (mol m^{-2} yr^{-1}) and decomposition of surface pCO_2 changes (ppm) into their **b,** DIC and alkalinity and **c,** SST and SSS components (Methods). Anomalies are at year 50 compared to the control run.

Core name	Latitude (°N)	Longitude (°E)	Depth (m)	Ref.	LGM δ^{13}C (‰)	HS1 δ^{13}C (‰)	$\Delta\delta^{13}$C (‰)	$\Delta\delta^{13}C_{av}$ (‰)
**Atlantic**								
ENAM93-21	62.44	-3.59	1020	18	0.12	-1.68	-1.8	-1.455
Rapid-10-1P	62.98	-17.59	1237	19	1.42	0.31	-1.11	
EW9302-26GGC	62.32	-21.46	1450	20	1.6	0.44	-1.16	-1.195
EW9302-25GGC	62.06	-21.47	1523	20	1.55	0.32	-1.23	
NEAP4k	61.29	-24.17	1627	19, 20	1.62	0.52	-1.1	-1.11
EW9302-24GGC	62	-21.67	1629	20	1.53	0.25	-1.28	
ODP 984	61	-24	1648	19, 20	1.6	0.64	-0.96	
Rapid-15-4P	63.29	-17.13	2133	20	0.886	0.38	-0.506	-0.623
RAPiD-17-5P	61.48	-19.54	2303	20	0.6	-0.14	-0.74	
BOFS 17K	58	-16.5	1150	21	1.55	1.13	-0.42	-0.42
KN166-14-JPC-13	53.06	-33.53	3082	22	0.36	0.09	-0.27	-0.27
BOFS 10K	54.7	-20.7	2777	21	0.37	0.69	0.32	0.32
IODP U1308	49.88	-24.24	3883	20	0.32	-0.27	-0.59	-0.59
BOFS 5K	50.7	-21.9	3547	21	0.41667	0.41	-0.00667	-0.00667
BOFS 8K	52.5	-22.1	4045	21	0.1583	0	-0.1583	-0.1583
NA87-22	55.29	-14.42	2161	18	0.78	0.11	-0.67	-0.67
CH69-K09	41.45	-47.21	4100	18	0.07	-0.34	-0.41	-0.41
MD95-2037	37.05	-32.01	2159	18	1.08	0.12	-0.96	-0.96
MD95-2040	40.58	-9.86	2465	23	0.46	-0.06	-0.52	-0.52
MD95-2042	37.8	-10.17	3146	24	0.19	0.04	-0.15	-0.298
MD99-2334K	37.48	-10.1	3146	25	0.246	-0.2	-0.446	
MD95-2039	40.58	-10.35	3381	26	0.2	-0.16	-0.36	-0.36
GIK12328-4	21.15	-18.57	2798	27	0.44	-0.09	-0.53	-0.53
M35003	12.5	-61.15	1299	18	1.25	0.45	-0.8	-0.75
MD99-2198	12.09	-61.23	1268	28	1.3	0.6	-0.7	
VM28-122	12	-79	1800	29, 30	1.13	0.42	-0.71	-0.71

Supplementary Table 2: **Benthic δ^{13}C** (‰) as measured in marine sediment cores during the LGM (21–19ka) and during HS1 (16.5–15.5 ka); Benthic δ^{13}C anomaly during HS1 compared to the LGM and $\Delta\delta^{13}C_{av}$ the final benthic δ^{13}C anomaly, in some cases reflecting an average of nearby cores.

Core name	Latitude (°N)	Longitude (°E)	Depth (m)	Ref.	LGM δ^{13}C (‰)	HS1 δ^{13}C (‰)	$\Delta\delta^{13}$C (‰)	$\Delta\delta^{13}C_{av}$ (‰)
**Atlantic - continued**								
GeoB1105-4	-1.67	-12.43	3225	31	0.24	-0.18	-0.42	-0.42
GeoB1115-4	-3.56	-12.56	2921	31	0.1	-0.17	-0.27	-0.27
GeoB1117	-3.82	-14.9	3984	31	0	-0.77	-0.77	-0.77
GeoB1711	-23.32	12.38	1967	18	0.23	0.16	-0.07	-0.07
KNR159-5-14GGC	-26.68	-46.5	441	19	1.17	1.139	-0.031	-0.031
KNR159-5-90GGC	-27.35	-46.63	1105	19	0.3	0.7658	0.4658	0.4658
KNR159-5-36GGc	-27.27	-46.47	1268	19	0.5507	0.4476	-0.1031	-0.1031
KNR159-5-17JPC	-27.7	-46.48	1627	19	0.9958	0.5251	-0.4707	
KNR159-5-78GGC	-27.48	-46.33	1820	19	1.0159	0.5212	-0.4947	-0.4699
KNR159-5-33GGC	-27.57	-46.18	2082	19	0.8	0.3557	-0.4443	
KNR159-5-42JPC	-27.76	-46.63	2296	19	0.484	0.2	-0.284	-0.1925
KNR159-5-73GGC	-27.89	-46.04	2397	19	0.379	0.278	-0.101	
KNR159-5-30GGC	-28.13	-46.04	2500	19	0.4623	0.4078	-0.0545	-0.0545
KNR159-5-63GGC	-28.36	-45.84	2732	19	0.3142	-0.128	-0.4422	-0.4061
KNR159-5-20JPC	-28.64	-45.54	2951	19	0.2048	-0.1652	-0.37	
KNR159-5-125GGC	-29.52	-45.75	3589	19	0.0555	-0.078	-0.1335	-0.1335
KNR159-5-22GGC	-29.79	-43.59	3924	19	-0.291	0.0246	0.3156	0.3156
MD02-2594	-34.43	17.2	2440	32	0.3	0.05	-0.25	-0.25
MD02-2588	-41.33	25.83	2907	33	0.03	0.33	0.3	0.3
TNO57-21	-41	7.8	4981	34	-0.99	-0.4	0.59	0.59
ODP1089	-40.56	9.54	4621	35, 36	-1.07	-0.43	0.64	0.575
RC11-83	-41.36	9.48	4718	36, 37	-0.93	-0.42	0.51	
MD07-3076Q	-44.09	-14	3770	18	-1.05	-1.05	0	0

Supplementary Table 2 - continued: **Benthic δ^{13}C** (‰) as measured in marine sediment cores. Negative latitudes denote degree south.

Core name	Latitude (°N)	Longitude (°E)	Depth (m)	Ref.	LGM δ^{13}C (‰)	HS1 δ^{13}C (‰)	$\Delta\delta^{13}$C (‰)
**Pacific**							
SO201-2-101KL	58.88	170.69	630	38	-0.11	0.095	0.205
MD02-2489	54.39	-148.92	3640	39	-0.52	-0.28	0.24
W8709A-13PC	49.72	168.3	2393	40	-0.55	-0.45	0.1
V19-4 GGC37	50.42	167.73	3300	41	-0.3	-0.45	-0.15
SO50-31KL	18.45	115.52	3360	42	-0.25	-0.43	-0.18
MD02-2529	8.12	-84.07	1619	43	0	0	0
MW91-9 GGC15	0	158	2310	44	-0.14	0.08	0.22
MW91-9 GGC48	0	161	3400	44	-0.26	-0.18	0.08
FR1/97 GC-12	-23.34	153.47	991	45	0.79	0.97	0.18
RR0503-87	-36	177	663	46	1.2	1	-0.2
RR0503-79	-36	177	1165	46	0.5	0.95	0.45
RR0503-83	-36	177	1627	46	-0.1	0.1	0.2
H214	-36	177	2045	46	-0.2	0	0.2
RR0503-125	-36	177	2541	46	-0.4	-0.15	0.25
Z2112	-34	168	2858	46	-0.2	0	0.2
RR0503-41	-40	177	3836	46	0	0	0
RS147-GC07	-45	148	3300	46	-0.2	0	0.2
SO213/2-84-1	-45.13	174.58	972	47	0.51	1.17	0.66
SO136-003GC	-42.30	169.88	944	47	0.84	1.26	0.42
MD06-2986	-43.45	167.9	1477	47	0.22	0.49	0.27
SO213/2-82-1	-45.78	176.6	2066	47	0	0.29	0.29
MD97-2120	-45.53	174.93	1210	48	0.3	0.8	0.5
MD07-3088	-46	-75	1536	49	-0.22	0.34	0.56
E11-2	-56.04	-115.05	3109	34	-0.45	-0.33	0.12

Supplementary Table 2 - continued: **Benthic δ^{13}C** (‰) as measured in marine sediment cores. Negative latitudes denote degree south.

Core name	Latitude (°N)	Longitude (°E)	Depth (m)	Ref.	LGM Vent. age (years)	HS1 Vent. age (years)	ΔVent. age (years)
**Atlantic**							
RAPiD-17-5P	61.29	-19.32	2303	50	2600	3952	1352
MD99-2334K	37.48	-10.1	3146	51	3094	3233	139
MD03-2707	2.3	9.24	1295	52	1147	1342	195
GS07-150-17/1GC-A	-4.13	-37.05	1000	53	986	1318	332
Corals	14.89	-48.15	795	5	1129	927	-202
Corals	9.22	-21.3	1080	5	1066	1111	45
Corals	9.21	-21.3	1366	5	1265	1271	6
Corals	-60	-58:-70	800–1500	4, 5	2030	1618	-411
KNR-159-5-78GGC	-27.48	-46.33	1829	19	1090	1430	340
MD07-3076CQ	-44.4	-14.12	3770	54	3606	2615	-991

Supplementary Table 3: **Ventilation age** (years) as measured in marine sediment cores during the LGM (21–19ka), HS1 (16.5–15.5 ka) and the HS1 anomaly compared to the LGM. Negative latitudes denote degree S. Ventilation ages were calculated by $\tau = \frac{1}{\lambda}.ln(\frac{\Delta^{14}C_{atm}+1000}{\Delta^{14}C_{bf}+1000})$, with $\Delta^{14}C_{atm}$ the contemporaneous IntCal 2013 atmospheric Δ^{14}C value[6] and $\Delta^{14}C_{bf}$ the benthic data.

Core name	Latitude (°N)	Longitude (°E)	Depth (m)	Ref.	LGM Vent. age (years)	HS1 Vent. age (years)	ΔVent. age (years)
**Pacific**							
PS75-104-1	-44.8	174.5	835	55	1739	1609	-130
SO213/2-76-2	-46.2	-178.03	4339	55	2662	2344	-318
SO213/2-79-2	-45.8	179.6	3142	55	3822	2734	-1088
SO213/2-82-1	-45.8	176.6	2066	55	4675	2191	-2484
MD07-3088	-46	-75	1536	49	2185	1996	-189
RR0503-64	-37.25	177	651	56	1070	1260	190
MD97-2120	-43.32	175	1210	56	1006	1050	44
SO161-SL122	-36	-73	1000	57	1222	632	-589
MV99-GC31/PC08	23.5	-111.6	705	58, 59	1848	2425	577
TR163-31	-3.62	-83.97	3210	59	3616	1490	-2126
MD01-2386	1	130	2800	59	2342	1946	-3967
KT89-18-P4	32.15	133.9	2700	59	2581	1726	-855
ODP893	34.29	-120.04	588	59	1295	1309	14
ODP1019	41.68	-124.93	980	59	2099	2028	-72
W8709A-13PC	42.12	-125.75	2712	59	2674	2716	42
GH02-1030	42.23	144.21	1212	59	2427	1902	-525
ODP887	54.37	-148.45	3467	59, 60	2783	2756	-28
MR01K03-PC4/PC5	41.12	142.4	1366	59	2587	1547	-1041
MD02-2489	54.39	-149	3640	61, 62	3306	1727	-1579
MD01-2416	51.27	167.73	2317	61	4185	3472	-713

Supplementary Table 3 - continued: **Ventilation age** (years) as measured in marine sediment cores during the LGM (21–19ka), HS1 (16.5–15.5 ka) and the HS1 anomaly compared to the LGM.

1. Shakun, J. *et al.* Global warming preceded by carbon dioxide concentrations during the last deglaciation. *Nature* **484**, 49–55 (2012).

2. Stríkis, N. M. *et al.* Timing and structure of Mega-SACZ events during Heinrich Stadial 1. *Geophysical Research Letters* **42**, 5477–5484A (2015). URL http://dx.doi.org/10.1002/2015GL064048. 2015GL064048.

3. Zhang, W. *et al.* A detailed East Asian monsoon history surrounding the 'Mystery Interval' derived from three Chinese speleothem records. *Quaternary Research* **82**, 154 – 163 (2014).

4. Burke, A. & Robinson, L. The Southern Ocean's role in carbon exchange during the last deglaciation. *Science* **335**, 557–561 (2012).

5. Chen, T. *et al.* Synchronous centennial abrupt events in the ocean and atmosphere during the last deglaciation. *Science* **349**, 1537–1541 (2015).

6. Reimer, P. *et al.* IntCal13 and Marine13 radiocarbon age calibration curves, 0-50,000 years cal BP. *Radiocarbon* **55**, 1869–1887 (2013).

7. Reimer, P. *et al.* IntCal09 and Marine09 radiocarbon age calibration curves, 0-50,000 years cal BP. *Radiocarbon* **51**, 1111–1150 (2009).

8. Reimer, P. *et al.* IntCal04 terrestrial radiocarbon age calibration, 0-26 cal kyr B.P. *Radiocarbon* **46**, 1111–1150 (2004).

9. Christopher, B. R. *et al.* A Complete Terrestrial Radiocarbon Record for 11.2 to 52.8 kyr B.P. *Science* **338**, 370–374 (2012).

10. Hughen, K., Eglinton, T., Xu, L. & Makou, M. Abrupt tropical vegetation response to rapid climate changes. *Science* **304**, 1955–1959 (2004).

11. Hughen, K., Southon, J., Lehman, S., Bertrand, C. & Turnbull, J. Marine-derived 14C calibration and activity record for the past 50,000 years updated from the Cariaco Basin. *Quaternary Science Reviews* **25**, 3216 – 3227 (2006).

12. Southon, J., Noronha, A. L., Cheng, H., Edwards, R. L. & Wang, Y. A high-resolution record of atmospheric 14C based on Hulu Cave speleothem H82. *Quaternary Science Reviews* **33**, 32 – 41 (2012).

13. Hain, M. P., Sigman, D. M. & Haug, G. H. Distinct roles of the Southern Ocean and North Atlantic in the deglacial atmospheric radiocarbon decline. *Earth and Planetary Science Letters* **394**, 198 – 208 (2014).

14. Marcott, S. *et al.* Centennial-scale changes in the global carbon cycle during the last deglaciation. *Nature* 616–619 (2014).

15. Bauska, T. *et al.* Carbon isotopes characterize rapid changes in atmospheric carbon dioxide during the last deglaciation. *Proceedings National Academy Sciences* **113**, 3465–3470 (2016).

16. Parrenin, F. *et al.* Synchronous change of atmospheric CO_2 and Antarctic temperature during the last deglacial warming. *Science* **339**, 1060–1063 (2013).

17. Olsen, A. *et al.* The Global Ocean Data Analysis Project version 2 (GLODAPv2) - an internally consistent data product for the world ocean. *Earth System Science Data* **8**, 297–323 (2016).

18. Waelbroeck, C. *et al.* The timing of deglacial circulation changes in the Atlantic. *Paleoceanography* **26**, PA3213 (2011).

19. Lund, D., Tessin, A., Hoffman, J. & Schmittner, A. Southwest Atlantic water mass evolution during the last deglaciation. *Paleoceanography* **30**, 477 – 494 (2015).

20. Oppo, D. W., Curry, W. B. & McManus, J. F. What do benthic δ^{13}C and δ^{18}O data tell us about Atlantic circulation during Heinrich Stadial 1? *Paleoceanography* **30**, 353–368 (2015). URL http://dx.doi.org/10.1002/2014PA002667.

21. Yu, J., Elderfield, H. & Piotrowski, A. Seawater carbonate ion-δ^{13}C systematics and application to glacial-interglacial North Atlantic ocean circulation. *Earth and Planetary Science Letters* **271**, 209–220 (2008).

22. Hodell, D., Evans, H., Channell, J. & Curtis, J. Phase relationships of North Atlantic ice-rafted debris and surface-deep climate proxies during the last glacial period. *Quat. Sci. rev.* **29**, 3875–3886 (2010).

23. Voelker, A. H. L. & de Abreu, L. *A Review of Abrupt Climate Change Events in the Northeastern Atlantic Ocean (Iberian Margin): Latitudinal, Longitudinal, and Vertical Gradients*, 15–37 (American Geophysical Union, 2013). URL http://dx.doi.org/10.1029/2010GM001021.

24. Shackleton, N., Hall, M. & Vincent, E. Phase relationships between millennial-scale events 64,000-24,000 years ago. *Paleoceanography* **15**, 565–569 (2000).

25. Skinner, L. & Shackleton, N. Rapid transient changes in norteast Atlantic deep water ventilation age across Termination I. *Paleoceanography* **19**, PA2005 (2004).

26. Schönfeld, J., Zahn, R. & de Abreu, L. Surface and deep water response to rapid climate changes at the Western Iberian Margin. *Global Planet. Change* **36**, 237–264 (2003).

27. Sarnthein, M. *et al.* Changes in east Atlantic deep water circulation over the last 30,000 years: Eight time slice reconstructions. *Paleoceanography* **9**, 209–269 (1994).

28. Pahnke, K., Goldstein, S. & Hemming, S. Abrupt changes in Antarctic Intermediate Water circulation over the past 25,000 years. *Nature Geoscience* **1**, 870–874 (2008).

29. Yu, J., Foster, G., Elderfield, H., Broecker, W. & Clark, E. An evaluation of benthic foraminiferal b/ca and δ^{11}b for deep ocean carbonate ion and ph reconstructions. *Earth and Planet. Sci. Lett.* **293**, 114–120 (2010).

30. Oppo, D. & Fairbanks, R. Variability in the deep and intermediate water circulation of the Atlantic $\Delta^{14}CO_2$ Ocean during the past 25,000 years: Northern hemisphere modulation of the Southern Ocean. *Earth and Planetary Science Letters* **86**, 1–15 (1987).

31. Bickert, T. & Mackensen, A. *The South Atlantic in the late Quaternary: Reconstruction of material budgets and current systems*, chap. Last Glacial to Holocene Changes in South Atlantic deep water circulation, 671–695 (Springer-Verlag Berlin, 2004).

32. Negre, C. *et al.* Reversed flow of Atlantic deep water during the Last Glacial Maximum. *Nature* **468**, 84 – 88 (2010).

33. Ziegler, M., Diz, P., Hall, I. & Zahn, R. Millennial-scale changes in atmospheric CO2 levels linked to the Southern Ocean carbon isotope gradient and dust flux. *Nature Geoscience* **6**, 457–461 (2013).

34. Ninnemann, U. & Charles, C. Changes in the mode of Southern Ocean circulation over the last glacial cycle revealed by foraminiferal stable isotopic variability. *Earth and Planetary Science Letters* **201**, 383–396 (2002).

35. Hodell, D., Venz, K., Charles, C. & Ninnemann, U. Pleistocene vertical carbon isotope and carbonate gradients in the South Atlantic sector of the Southern Ocean. *Geochem. Geophys. Geosys.* **4**, 1004 (2003).

36. Barker, S. & Diz, P. Timing of the descent into the last Ice Age determined by the bipolar seesaw. *Paleoceanography* **29**, 489–507 (2014).

37. Charles, C., Lynch-Stieglitz, J., Ninnemann, U. & Fairbanks, R. Climate connections between the hemispheres revealed by deep sea sediment core/ice core correlations. *Earth and Planetary Science Letters* **142**, 19–27 (1996).

38. Max, L. *et al.* Evidence for enhanced convection of north pacific intermediate water to the low-latitude pacific under glacial conditions. *Paleoceanography* **32**, 41–55 (2017). URL `http://dx.doi.org/10.1002/2016PA002994`. 2016PA002994.

39. Gebhardt, H. *et al.* Paleonutrient and productivity records from the subarctic North Pacific for Pleistocene glacial terminations I to V. *Paleoceanography* **23**, PA4212 (2008).

40. Lund, D. C. & Mix, A. C. Millennial-scale deep water oscillations: Reflections of the north atlantic in the deep pacific from 10 to 60 ka. *Paleoceanography* **13**, 10–19 (1998). URL http://dx.doi.org/10.1029/97PA02984.

41. Keigwin, L. D. Glacial-age hydrography of the far northwest Pacific Ocean. *Paleoceanography* **13**, 323–339 (1998). URL http://dx.doi.org/10.1029/98PA00874.

42. Wei, G., Wang, C., Lee, M. & Wei, K. High-resolution benthic foraminifer δ^{13}C records in the South China Sea during the last 150 ka. *Marine Geol.* **232**, 227–235 (2006).

43. Leduc, G., Vidal, L., Tachikawa, K. & Bard, E. Changes in Eastern Pacific ocean ventilation at intermediate depth over the last 150 kyr B.P. *Earth and Planetary Science Letters* **298**, 217 – 228 (2010).

44. Yu, J. *et al.* Loss of carbon from the deep sea since the Last Glacial Maximum. *Science* **330**, 1084–1087 (2010).

45. Bostock, H., Opdyke, B., Gagan, M. & Fifield, L. Carbon isotope evidence for changes in Antarctic Intermediate Water circulation and ocean ventilation in the southwest Pacific during the last deglaciation. *Paleoceanography* **19**, PA4013 (2004).

46. Sikes, E., Elmore, A., Allen, K., Cook, M. & Guilderson, T. Glacial water mass structure and rapid δ^{18}O and δ^{13}C changes during the last glacial termination in the Southwest Pacific. *Earth and Planetary Science Letters* **456**, 87–97 (2016).

47. Ronge, T. A. *et al.* Pushing the boundaries: Glacial/interglacial variability of intermediate and deep waters in the southwest pacific over the last 350,000 years. *Paleoceanography* **30**, 23–38 (2015). URL `http://dx.doi.org/10.1002/2014PA002727`. 2014PA002727.

48. Pahnke, K. & Zahn, R. Southern Hemisphere water mass conversion linked with North Atlantic climate variability. *Science* **307**, 1741–1746 (2005).

49. Siani, G. *et al.* Carbon isotope records reveal precise timing of enhanced Southern Ocean upwelling during the last deglaciation. *Nature Communications* **4**, 2758 (2013).

50. Thornalley, D., Barker, S., Broecker, W., Elderfield, H. & McCave, I. The deglacial evolution of North Atlantic Deep Convection. *Science* **331**, 202–205 (2011).

51. Skinner, L., Waelbroeck, C., Scrivner, A. & Fallon, S. Radiocarbon evidence for alternating northern and southern sources of ventilation of the deep Atlantic carbon pool during the last deglaciation. *Proceedings of the National Academy of Science* **111**, 5480–5484 (2014).

52. Weldeab, S., Friedrich, T., Timmermann, A. & Schneider, R. Strong middepth warming and weak radiocarbon imprints in the equatorial Atlantic during Heinrich 1 and Younger Dryas. *Paleoceanography* **31**, 1070 – 1082 (2016).

53. Freeman, E. *et al.* An AtlanticPacific ventilation seesaw across the last deglaciation. *Earth and Planetary Science Letters* **424**, 237–244 (2015).

54. Skinner, L., Fallon, S., Waelbroeck, C., Michel, E. & Barker, S. Ventilation of the deep Southern Ocean and deglacial CO_2 rise. *Science* **328**, 1147–1151 (2010).

55. Ronge, T. *et al.* Radiocarbon constraints on the extent and evolution of the South Pacific glacial carbon pool. *Nature Communications* **7**, 11487 (2016).

56. Rose, K. *et al.* Upper-ocean-to-atmosphere radiocarbon offsets imply fast deglacial carbon dioxide release. *Nature* **466**, 1093 – 1097 (2010).

57. Pol-Holz, R. D., Keigwin, L., Southon, J., Hebbeln, D. & Mohtadi, M. No signature of abyssal carbon in intermediate waters off Chile during deglaciation. *Nature Geoscience* **3**, 192–195 (2010).

58. Marchitto, T., Lehman, S., Ortiz, J., Flückiger, J. & van Geen, A. Marine radiocarbon evidence for the mechanism of deglacial atmospheric CO_2 rise. *Science* **316**, 1456–1459 (2007).

59. Okazaki, Y. *et al.* Deep water formation in the North Pacific during the Last Glacial termination. *Science* **329**, 200–204 (2010).

60. Galbraith, E. *et al.* Carbon dioxide release from the North Pacific abyss during the last deglaciation. *Nature* **449**, 890–894 (2007).

61. Sarnthein, M., Schneider, B. & Grootes, P. Peak glacial ^{14}C ventilation ages suggest major draw-down of carbon into the abyssal ocean. *Climate of the Past* **9**, 2595–2614 (2013).

62. Rae, J. *et al.* Deep water formation in the North Pacific and deglacial CO_2 rise. *Paleoceanography* **29**, 645–667 (2014).
